# Specific Biological Features of Adipose Tissue, and Their Impact on HIV Persistence

**DOI:** 10.3389/fmicb.2019.02837

**Published:** 2019-12-17

**Authors:** Christine Bourgeois, Jennifer Gorwood, Aurélie Barrail-Tran, Claire Lagathu, Jacqueline Capeau, Delphine Desjardins, Roger Le Grand, Abderaouf Damouche, Véronique Béréziat, Olivier Lambotte

**Affiliations:** ^1^Center for Immunology of Viral Infections and Autoimmune Diseases, IDMIT Department, IBFJ, CEA, Université Paris Sud, INSERM U1184, Fontenay-aux-Roses, France; ^2^INSERM UMR_S 938, Centre de Recherche Saint-Antoine, Institut Hospitalo-Universitaire de Cardio-Métabolisme et Nutrition (ICAN), Sorbonne Université, Paris, France; ^3^AP-HP, Service de Médecine Interne et Immunologie Clinique, Hôpital Bicêtre, Groupe Hospitalier Universitaire Paris Sud, Le Kremlin-Bicêtre, France

**Keywords:** adipose tissue, fat, HIV, infectious disease, resident memory T cells, T lymphocyte, reservoir, immune response

## Abstract

Although white AT can contribute to anti-infectious immune responses, it can also be targeted and perturbed by pathogens. The AT’s immune involvement is primarily due to strong pro-inflammatory responses (with both local and paracrine effects), and the large number of fat-resident macrophages. Adipocytes also exert direct antimicrobial responses. In recent years, it has been found that memory T cells accumulate in AT, where they provide efficient secondary responses against viral pathogens. These observations have prompted researchers to re-evaluate the links between obesity and susceptibility to infections. In contrast, AT serves as a reservoir for several persistence pathogens, such as human adenovirus Ad-36, *Trypanosoma gondii*, *Mycobacterium tuberculosis*, influenza A virus, and cytomegalovirus (CMV). The presence and persistence of bacterial DNA in AT has led to the concept of a tissue-specific microbiota. The unexpected coexistence of immune cells and pathogens within the specific AT environment is intriguing, and its impact on anti-infectious immune responses requires further evaluation. AT has been recently identified as a site of HIV persistence. In the context of HIV infection, AT is targeted by both the virus and the antiretroviral drugs. AT’s intrinsic metabolic features, large overall mass, and wide distribution make it a major tissue reservoir, and one that may contribute to the pathophysiology of chronic HIV infections. Here, we review the immune, metabolic, viral, and pharmacological aspects that contribute to HIV persistence in AT. We also evaluate the respective impacts of both intrinsic and HIV-induced factors on AT’s involvement as a viral reservoir. Lastly, we examine the potential consequences of HIV persistence on the metabolic and immune activities of AT.

## Introduction

The three types of adipose tissue (AT: brown, white, and beige) each have specific functional properties (Cinti, 2012, 2018). Unless otherwise stated, the term “AT” will henceforth refer to white AT. The biology of AT is complex; this tissue exerts a range of metabolic, regenerative, immune, and other specific functions (Britton and Fox, 2011; Guerrero-Juarez and Plikus, 2018). The immune potential of AT is currently under renewed scrutiny. Historically, the strong pro-inflammatory responses within AT (exerting both local and paracrine effects), and the high number of fat-resident macrophages suggested that AT contributed to anti-infectious innate immune responses. The presence and persistence of bacterial DNA further suggests that a broad range of pathogens persists in AT (Burcelin et al., 2013). AT is a site of HIV persistence and appears as a crucial cofactor in both viral persistence and chronic immune activation/inflammation during HIV infection (Damouche et al., 2015; Gorwood et al., 2019). Concomitantly, studies on the AT’s immune compartment have highlighted the non-negligible contribution of resident immune cells [and particularly AT memory (Trm) CD8 T cells] to anti-infectious responses (Han et al., 2017). Here, we first review AT’s specific properties (independently of HIV infection) and, notably, its contribution to anti-infectious immune responses. We then discuss the changes in AT induced by HIV infection and/or antiretroviral therapy (ART). Lastly, we consider the potential consequences of HIV infection on the metabolic and immune functions of AT.

## Specific Features of Adipose Tissue

### The Anatomic and Functional Heterogeneity of Adipose Tissue

**FIGURE 1:**
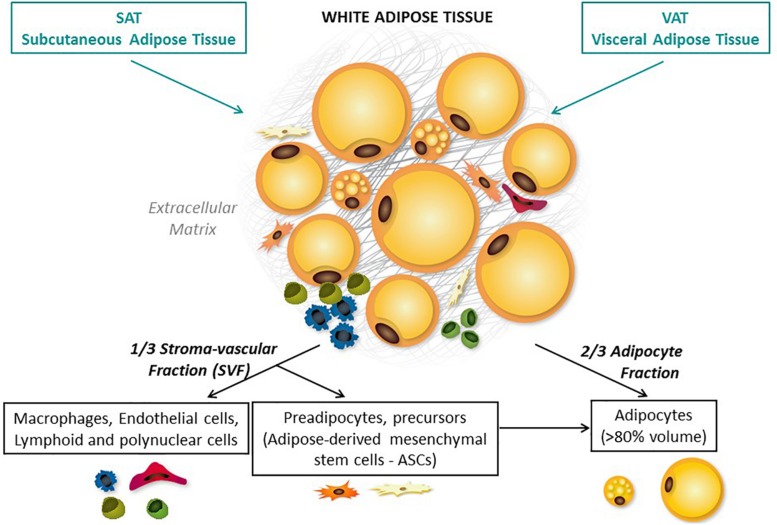
Adipose tissue distribution and composition. Adipose tissue is composed of two cell fractions that can be easily separated through collagenase digestion: the adipocytes and the stromal vascular fraction (SVF), both surrounded by extracellular matrix (ECM). All these three compartments are responsible for the pleiotropic roles of AT. Adipocytes are the main cellular component crucial for both energy storage and endocrine activity. The other cell type that are present are precursors (such as adipose-derived mesenchymal stem cells – ASCs), fibroblasts, vascular cells, and immune cells. AT is distributed across a large number of discrete anatomic sites (Shen et al., 2003; Lee et al., 2013). Subcutaneous AT (SAT, accounting for over 80% of total body fat) and visceral AT (VAT) are the best-studied depots.

In healthy males and females, AT accounts for, respectively, 15 and 25% of the body weight, and is distributed across a large number of discrete anatomic sites (Shen et al., 2003; Lee et al., 2013; Table 1). Subcutaneous AT (SAT, accounting for over 80% of total body fat) and visceral AT (VAT) are the best-studied depots (Figure 1). The classification of the various types of AT reflects differences in the tissues’ ontogeny (Chau et al., 2014) and metabolic profiles. SAT and VAT differ with regard to their metabolic activity and immune cell content (Wajchenberg, 2000; Fain et al., 2004; Ibrahim, 2009; Madani et al., 2009; Altintas et al., 2011). Furthermore, SAT can be divided into superficial SAT and deep SAT, which again differ with regard to their metabolic properties (Smith et al., 2001). Likewise, VAT depots can be subclassified as omental fat (protective layer surrounding the intestine), retroperitoneal fat (near the kidney), and mesenteric fat (close to the intestine), which again have different activities (Wronska and Kmiec, 2012). In addition to SAT and VAT, AT is present in multiple locations, and may exhibit even more specialized functions related to the associated tissue. Dermal AT (dAT) is a specialized adipose depot that is distinct from SAT. Lastly, perivascular AT (pAT) has a role in the homeostasis of the cardiovascular system (Cheng et al., 2018).

**TABLE 1:** The anatomical and functional heterogeneity of adipose tissues.

**Anatomical location/Sublocation**	**Functions**	**References**
**Major metabolic sites**		
Subcutaneous AT (SAT)	Metabolic activity	Lee et al., 2013; Chau et al., 2014
Superficial SAT		Smith et al., 2001
Deep SAT		
Visceral AT (VAT)	More active metabolically More strongly associated with metabolic diseases	Wronska and Kmiec, 2012
Omental		
Retroperitoneal		
Mesenteric		
**Adipose tissue associated with primary and secondary immune sites**		
Bone marrow AT (MAT)	Contributing to hematopoiesis Deleterious in excess	Hardouin et al., 2014; Scheller et al., 2016
AT embedding LN	Delivering nutrients to immune cells Favoring immune responses	Knight, 2008
Thymic AT	Associated to age-associated thymic involution and the loss of thymic function	Coín Aragüez et al., 2013
**Additional specialized adipose tissue**		
Dermal AT (dAT)	– Wound healing, hair follicle cycling, thermoregulation – Immune responses	Chase et al., 1953; Moffat, 1968; Alexander et al., 2015; Guerrero-Juarez and Plikus, 2018
Perivascular AT (pAT)	– Vascular homeostasis	Rajsheker et al., 2010; Britton and Fox, 2011; Szasz and Webb, 2012; Gu et al., 2019

Adipose tissue can also surround lymphoid structures [notably lymph nodes (LNs)] or even infiltrate them [e.g., the bone marrow (BM) and thymus]. The physiologic impact of AT also differs from one lymphoid site to another. For example, the infiltration of fat into the thymus is always associated with age-associated thymic involution and the loss of thymic function (Hale, 2004; Coín Aragüez et al., 2013), whereas fat infiltration into the BM (the third largest fat depot after SAT and VAT) is a physiologic feature initially required for hematopoiesis. However, an age-related increase in fat infiltration into the BM is associated with defective hematopoiesis – suggesting that too much fat is harmful. The AT that surrounds the LNs (perinodal fat) does not appear to infiltrate them (Knight, 2008). Perinodal AT is thought to deliver nutrients (such as fatty acids) to immune cells; this prevents activated lymphocytes from competing for blood nutrients, and improves immune responses (Pond, 2002). Conversely, chronic stimulation of LNs also influences the cellular composition of the perinodal AT (Mattacks et al., 2003). Inducible lymphoid structures have been identified at mucosal sites (i.e., mucosal-associated lymphoid tissue) and also in AT: in addition to the milky spots (MSs) previously described in the omentum, fat-associated lymphoid clusters (FALCs) are found in mesenteric and pericardial AT (Beelen, 1991; Cruz-Migoni and Caamaño, 2016). In contrast to fat-embedded LNs, FALCs and MSs are found at points of direct contact between immune cells and metabolic cells (Moro et al., 2010). It is not yet clear whether MSs and FALCs are different immune clusters (they can differ in their composition and size) (Moro et al., 2010; Lolmède et al., 2011; Meza-Perez and Randall, 2017; Bénézech and Jackson-Jones, 2019), although both have immune functions (Rangel-Moreno et al., 2009; Bénézech and Jackson-Jones, 2019). Group 2 innate lymphoid cells (ILC2s) and B cells are crucial components of FALCs, since they coordinate local immune responses in fat depots and contribute to AT homeostasis (Bénézech and Jackson-Jones, 2019) and anti-infectious responses (Jones et al., 2015). These immune clusters provided the first evidence of a direct role of fat immune cells in anti-infectious responses, and also highlight the “regionalization” of AT. In fact, AT is a vascularized tissue that is organized into several lobular unit (Tang et al., 2008; Walker et al., 2008; Chi et al., 2018; Dichamp et al., 2019). These partitioned areas exhibit specific metabolic (and probably immune) activities. As a general rule, it is important to take account of AT’s heterogeneity on two levels (i.e., the lymphoid structure considered, and the region within each AT depot). This heterogeneity may be associated with differences in the interactions between metabolic and immune cells (Mahlakõiv et al., 2019).

From an immunologic point of view, AT is close to most of the physical barriers in the organism [i.e., the digestive tract, respiratory tract (Chen et al., 2019), and skin] and lymphoid tissues. The proximity between AT and the immune sites raises the question of whether AT contributes significantly to local immune responses after the first physical barrier or mucosa has been breached. In fact, AT may act both passively and actively as a second line of defense against microbial invasion. Given that the various AT depots also differ in their immune cell composition, they may also differ in their role in immune responses.

### Metabolic Functions, Plasticity, and Expandability of Adipose Tissue

#### Physiological Metabolic Plasticity

Adipose tissue was initially defined as a metabolic site; it constitutes the body’s major energy storage site and is also an endocrine tissue that directly modulates systemic lipid and glucose metabolism and insulin sensitivity. AT is composed of two cell fractions: the adipocytes that represent approximately 80% of the volume of AT and 60% of the total cell population, and the stromal vascular fraction (SVF), both surrounded by extracellular matrix (ECM) (Figure 1). All these three compartments are responsible for the pleiotropic roles of AT. In physiological conditions, AT must be viewed primary as a protective tissue that stores and prevents excessive exposure of other organs to fatty acids. One key feature of AT is its ability to adapt and expand in response to energy needs or surpluses thanks to its cellular heterogeneity. The response to metabolic cues is based on adipocyte hypertrophy (increase in adipocyte volume) and/or hyperplasia (increase in adipocyte number by recruitment and proliferation of precursor cells and adipogenesis). Adipocyte volume reflects the specific function of adipocyte to store energy in the form of lipids, and thus, the cell capacity to dramatically modulate its size in response to changes in energy balance. Lipid accumulation is determined by the balance between lipogenesis and lipolysis. This plasticity also includes vascular remodeling and ECM remodeling to allow adequate tissue expansion, oxygenation, and mobilization of nutrients (Pellegrinelli et al., 2016).

The adipocyte’s metabolic activity is primarily modulated by insulin. When the energy balance is positive, insulin favors adipogenesis, lipogenesis from non-lipid precursor such as glucose, and the uptake of circulating free fatty acids (FFAs) to synthesize triacylglycerols (TGs) that will accumulate within the lipid droplets. In response to insulin, adipocytes secrete the lipoprotein lipase (that process TG into FFA and glycerol phosphate), but also favor glucose and glycerol influx by translocation of glucose transporter type 4 (GLUT4) to the plasma membrane and upregulation of glycerol channels. At the opposite, in time of energy shortage, adipocytes release FFA back in the circulation during lipolysis through the action of different lipases. It results in the efflux of glycerol-phosphate and FFA that serve as a source of energy for most tissues. Hence, under physiological conditions, adipocytes adapt rapidly to metabolic changes. Regional variations in the metabolic activity of adipocytes have been observed in healthy subjects, especially in regards to regulation of lipolysis and lipogenesis.

**FIGURE 2:**
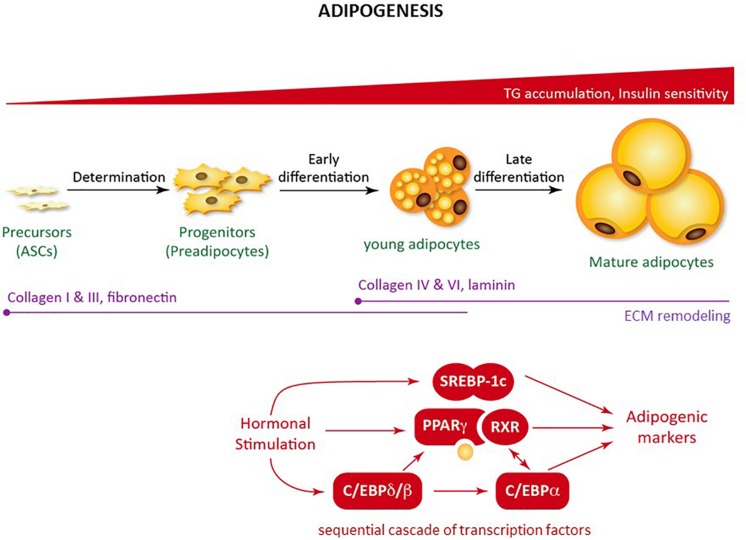
Adipogenesis. Schematic figure of the regulation of adipogenesis from mesenchymal precursors. Adipogenesis is a sequential cascade of transcription factors leading to lipid accumulation and the acquisition of insulin sensitivity. Adipogenesis also involves extracellular matrix remodeling.

Even in non-expanding AT, adipocytes are renewed frequently to compensate for adipocyte death, with a turnover of approximately 10% annually (Spalding et al., 2008), indicating that committed adipocyte progenitors/precursors exist within AT. Adipose-derived mesenchymal stem cells (ASCs), defined as mesenchymal stem cells (MSCs) with similar clinical potential as MSCs from BM, were discovered. ASCs were identified as multipotent cells capable of *in vitro* differentiation into adipocytes, osteoblasts, chondrocytes, and myoblasts (Rodriguez et al., 2005; Cawthorn et al., 2012). Adipocyte differentiation starts with a first phase called determination, during which a multipotent precursor (ASCs) commits itself to adipocyte lineage and becomes a preadipocyte, unable to differentiate into any other cell type. Adipogenic cascade and molecular regulation of adipogenesis requires the coordination of multiple regulatory and signaling pathways. During the second phase, called adipogenesis, the preadipocyte differentiates into a mature adipocyte that acquires all the machinery required for lipid transport and synthesis, insulin sensitivity, and adipokine secretion. Adipogenesis is characterized by cell cycle arrest and the induction of a transcriptional cascade: Peroxisome proliferator-activated receptor gamma (PPAR-γ), the master regulator of adipogenesis (Shao et al., 2016), several members of the CCAAT-enhancer binding protein (C/EBP) following a strict temporal regulation, C/EBPβ and C/EBPδ inducing C/EBPα (Rosen and MacDougald, 2006), and sterol regulatory element binding protein 1c (SREBP1c) (Peirce et al., 2014; Figure 2).

Studies of obesity have uncovered the presence of metabolically active immune cells within AT (Mathis et al., 2011). Under physiological conditions, immune cells in the AT [notably anti-inflammatory M2 macrophages (Morris et al., 2011) and CD4 regulatory T cells (Tregs) (Feuerer et al., 2009; Cipolletta, 2014; Kolodin et al., 2015)] ensure homeostasis by remodeling the ECM (Odegaard and Chawla, 2013), eliminating dying adipocytes, and limiting inflammation. Natural killer T (NKT) cells (Lynch et al., 2012, 2015), ILCs (Flach and Diefenbach, 2015; Lee et al., 2016; O’Sullivan et al., 2016; Boulenouar et al., 2017), and eosinophils (Wu et al., 2011; Lee E.-H. et al., 2018) have also been reported, although their net activity to metabolic homeostasis is subject to debate. In essence, all types of immune cell have now been found in AT, although the AT’s specific immune content appears to vary from one anatomic site to another. Furthermore, it is not clear whether the immune compartment of AT changes transiently in response to metabolic cues during the physiological cycle between fasting and satiety.

#### Defects in Adipose Tissue Expandability

When the AT’s buffering capacity is exceeded (as a consequence of sustained lipid/nutrient overload, for example), a wide range of disorders can result (de Ferranti and Mozaffarian, 2008): ectopic accumulation of lipids within the body, insulin resistance, and the associated metabolic complications (Lionetti et al., 2009) (such as obesity and type II diabetes). These disorders are associated with major changes in the metabolic and immune compartments of AT. Adipose hypertrophy and hyperplasia are both associated with intracellular dysfunction in adipocytes (molecular changes in the lipolytic and lipogenic pathways, endoplasmic reticulum, and mitochondrial stresses). In turn, these changes lead to the release of pro-inflammatory adipokines, free fatty acids, and inflammatory mediators that exacerbate the dysfunction in AT. Changes in macrophage polarization (Lumeng et al., 2007), a reduction in the proportion of Treg cells (Feuerer et al., 2009), and an accumulation of CD8 T cells (Nishimura et al., 2009b) have been reported in mice, and these immune alterations participate in the development of metabolic alterations within AT. In the context of obesity, the interaction between adipocytes and macrophages is crucial. Firstly, obesity is associated with the recruitment of macrophages to the AT – although the exact mechanism has yet to be characterized (Weisberg et al., 2003; Nishimura et al., 2009b). These macrophages produce pro-inflammatory cytokines, which in turn diminish the adipocytes’ insulin sensitivity [described for TNF-α in rodents (Xu et al., 2003)]. Lastly, insulin resistance impairs the hormone’s ability to suppress lipolysis (Bastard et al., 2006). The accumulation of large body of data on the critical interactions between metabolic cells and immune cells has prompted the emergence of a new field of research: immunometabolism.

### Adipose Tissue Senses and Sends Multiple Signals

**FIGURE 3:**
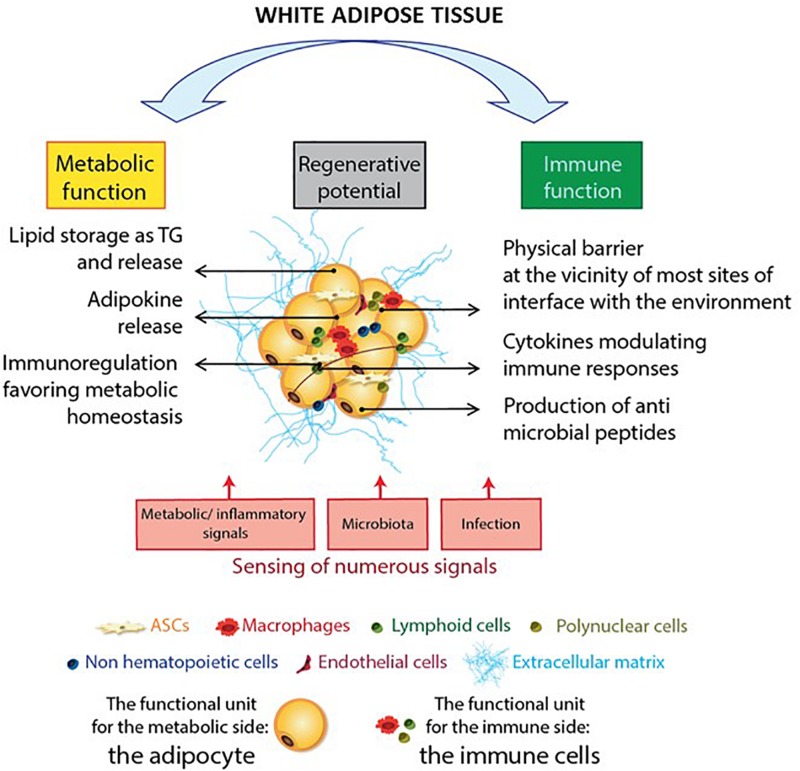
The adipose tissue as a sensor: the AT immune and metabolic sides. Adipose tissue has many functional activities, including metabolic functions, immune responses, and regeneration. The large, ubiquitous depots of AT provide local storage for stem cell progenitors, and innate and adaptive immune cells. The adipocyte is the functional unit in the AT’s metabolic activity. Immune cells ensure adipose tissue homeostasis by providing a controlled environment that favors adipogenesis and metabolic homeostasis. Interestingly, AT components are also involved in anti-infectious immune responses: adipocytes exert an anti-infectious response by producing antimicrobial peptides such as the cathelicidins while both innate and adaptive immune cells contribute to anti-infectious responses. Another important feature of AT is its high sensitivity to environmental cues, such as metabolic and inflammatory signals, the microbiota, and infectious episodes.

Adipose tissue sense a number of hormonal, metabolic, and/or inflammatory signals (Kim et al., 2014; Cousin et al., 2015), as well as neuronal signals (Chi et al., 2018) and infectious episodes (Casteilla et al., 2011). The main modulator of AT is the nutritional status. Studies of obesity have demonstrated that AT is extremely flexible; it can exert anti- or pro-inflammatory actions, depending on the metabolic context. AT also differs when comparing males and females (Poglio et al., 2010; Luche et al., 2015) due to hormonal influences. AT is also innervated by both sympathetic and parasympathetic systems, regulating AT growth (Engela et al., 2013). In turn, AT-derived signals regulate food intake and peripheral metabolism. The endocrine activity of AT is a pivotal pathway of interactions between AT and other organs. In that way, the brain–gut–AT axis is a significant communication pathway (Franquesa et al., 2015). Interestingly, germ-free animals have 40% less fat mass than mice raised under conventional microbial conditions (Puissant et al., 2005), suggesting a gut microbiota’s direct contribution to AT biology. Pioneering studies have demonstrated that lean and obese animals differ with regard to their gut microbiota. Microbial dysbiosis in obese animals may subsequently affect the efficiency of calorie harvesting from the diet, and calorie storage in AT (Puissant et al., 2005; Le Blanc and Mougiakakos, 2012; Luz-Crawford et al., 2013). Accordingly, certain strains of microbes appear to induce obesity (Prockop and Oh, 2012; Wang et al., 2014) and impair immune functions, which may increase the organism’s susceptibility to infections (Bornstein et al., 2000; Poglio et al., 2012; Lee Y.S. et al., 2018; Figure 3). Lastly, AT is also a site of accumulation of persistent organic pollutants and various lipophilic drugs (Kintscher et al., 2008) that makes AT a potential sensor of environmental toxicity.

### The Accumulation of Hematopoietic Stem and Precursor Cells and Mesenchymal Stem Cells in Adipose Tissue

In addition to AT’s involvement in metabolic homeostasis, this tissue also contains large numbers of both hematopoietic stem and precursor cells (HSPCs) and ASCs (Baglioni et al., 2009; Kim et al., 2014; Cousin et al., 2015). These cells confer AT with a high regenerative potential, which is currently being investigated for therapeutic purposes (Poglio et al., 2010; Casteilla et al., 2011; Luche et al., 2015). Importantly, ASCs enable tissue repair but also regulate inflammation, and their immunomodulatory properties have been extensively studied (Puissant et al., 2005; Le Blanc and Mougiakakos, 2012; Prockop and Oh, 2012; Engela et al., 2013; Luz-Crawford et al., 2013; Franquesa et al., 2015). ASCs appear to affect both innate and adaptive immunity. However, recent data suggest that this immunosuppressive profile may not be constitutive, and may depend on the pathophysiologic context (Wang et al., 2014). In contrast, data on HSPCs and the latter’s regenerative potential are scarce (Cousin et al., 2015). However, Poglio et al. (2010) demonstrated that AT is a reservoir of functional mast cell progenitors and these progenitors may renew innate immunity in AT but also support innate immunity in other organs (Poglio et al., 2012). AT may thus constitute an alternate source of immune precursors, which might be involved in the development of long-term, sustained immune responses.

### The Intrinsic Coexistence of Metabolic Cells and Immune Cells in Adipose Tissue: The Concept of Immunometabolism

**FIGURE 4:**
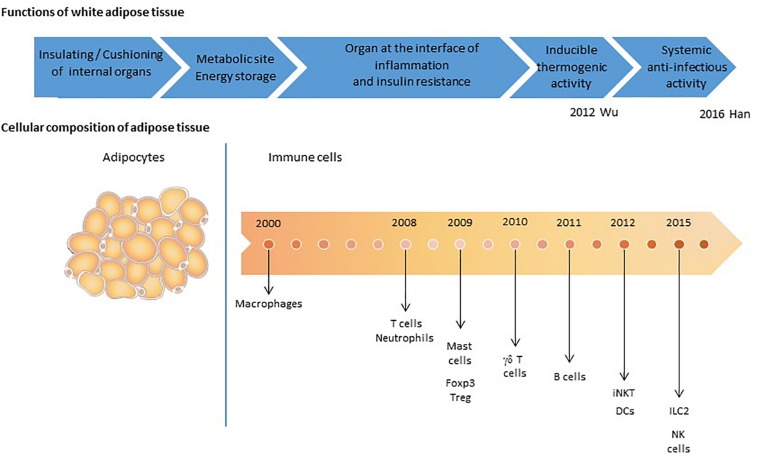
The detection of immune cells in adipose tissue: a time line. Adipose tissue was initially defined as a cushioning site, and subsequently as a metabolic site mainly composed of adipocytes. The AT’s immune function has emerged progressively. The first cell subset to be identified was the macrophage population (Bornstein et al., 2000; Weisberg et al., 2003; Xu et al., 2003), which contributes to the homeostasis of AT *via* the clearance of cellular debris, lipid buffering, and immune surveillance. T cell populations were then also described (Wu et al., 2007; Kintscher et al., 2008; Rausch et al., 2008), followed by neutrophils (Elgazar-Carmon et al., 2008), mast cells (Liu et al., 2009), regulatory T cells (Tregs) (Feuerer et al., 2009), B cells (Winer et al., 2011), eosinophils (Wu et al., 2011), dendritic cells (DCs) (Bertola et al., 2012), iNKT cells (Lynch et al., 2012), natural killer (NK) cells (Wensveen et al., 2015; Lee et al., 2016), and innate lymphoid cells (ILCs) (Brestoff et al., 2015; Lee et al., 2015; O’Sullivan et al., 2016). The cell types’ respective roles in local metabolic homeostasis have been documented, although data on their potential anti-infectious activities are scarce. The recent publication of Han et al. (2017) provide a significant evidence of the contribution of AT immune cells to anti-infective immune responses.

Immunometabolism refers to the interplay between immune processes and metabolic pathways (Mathis et al., 2011; Lee Y.S. et al., 2018). AT is the prototypic immunometabolic tissue in which immune and metabolic cells interact (Figure 3). The SVF contains a broad range of immune cells, such as macrophages [detected in the early 2000s (Bornstein et al., 2000; Weisberg et al., 2003)] and T lymphocytes (Wu et al., 2007; Kintscher et al., 2008). Characterization of the immune compartment in AT has confirmed or revealed the presence of DCs (Bertola et al., 2012), neutrophils (Elgazar-Carmon et al., 2008), eosinophils (Wu et al., 2011), mast cells (Liu et al., 2009), and (for the lymphoid compartment) NK cells (Boulenouar et al., 2017), invariant NKT cells (Lynch et al., 2012), B cells (Nishimura et al., 2013), γδT cells (Zúñiga et al., 2010), and ILCs (Ferrante, 2013; Figure 4). In healthy individuals, AT is mainly described as an anti-inflammatory site, due to the high proportion of cells with an immunosuppressive phenotype. Given that immune cells in AT and their role as metabbolic modulators have been exhaustively reviewed elsewhere (Nishimura et al., 2009a; Schaffler and Scholmerich, 2010; Lolmède et al., 2011; de Jong et al., 2014; Guzik et al., 2017), we shall focus on the AT-derived parameters that may be linked to anti-infectious properties.

Metabolic and immune cells obviously have different functional activities, and are also at opposite ends of the spectrum with regard to their metabolic strategies and energy needs. Whereas metabolic cells have large intracellular energy storage vesicles, immune cells mainly rely on external energy supplies. Indeed, immune cells and immune responses depend closely on external energy supplies. However, one can consider that this strategy is well suited to the rapid changes in metabolic status that accompany immune activation and proliferation.

### Summary of the Biological Features of Adipose Tissue

In brief, AT comprises many different depots, some of which make specific contributes to the homeostasis of various tissues (BM, skin, blood vessels, etc.). The main AT depots (SAT and VAT) are capable of integrating signals from various sources and modulating their biology accordingly (Figure 1). A depot’s functional properties depend on its location (SAT vs. VAT) and may be partitioned within a given location. The AT’s sensitivity to metabolic and inflammatory signals also influences the tissue’s immune cell composition and properties. In rodent models of obesity, massive changes in the composition of the SVF has been described. One might think that adipocytes’ metabolic plasticity is related to changes in the AT’s immune functions. In fact, these two phenomena are relatively disconnected. Immune plasticity appears to be rather associated with the loss of adipocyte plasticity and the lipid spillover that initiates local inflammation. The AT’s high sensitivity to environmental cues complicates studies of human samples. The highly heterogeneous metabolic context and history of infections in clinical trial participants constitute a major limitation.

## Adipose Tissue and Infections

As always in research on AT, the initial datasets emerged from studies of obesity. Interestingly, obesity and susceptibility to infections appears to have a two-way link, and AT has a key role in the dialog between metabolism and the immune system. On the one hand, it has been shown that infection impacts AT biology both indirectly (*via* the bystander effect of inflammation) and/or directly (*via* the impact of local pathogen persistence). These findings strengthen the “infectobesity” hypothesis, in which obesity has an infectious etiology. On the other hand, a growing body of evidence indicates that the obesity-induced disruption of AT direct influences the patients’ susceptibility to infection. AT therefore appears to contribute to anti-infectious immune responses and, at the same time, constitute a target for pathogens and a site at which infections induce perturbations. Both of these aspects will be reviewed below – firstly in contexts unrelated to HIV, and secondly in the specific context of HIV infection.

**FIGURE 5:**
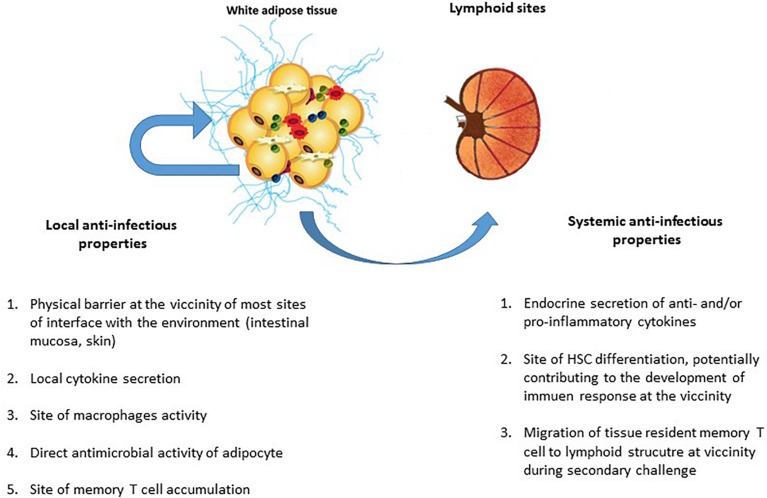
The local and systemic anti-infectious properties of adipose tissue. The AT’s contribution to anti-infectious immune responses takes several forms. In addition to AT’s local anti-infectious activity, AT-resident memory T cells contributed immune defense against pathogens (as in other non-lymphoid tissues). Although it was initially considered to be a local barrier that protect other tissues after a mucosal breach, AT may also contribute to effective secondary adaptive immune responses. The AT’s local contribution to immune responses to infection includes (i) the maintenance of an effective physical barrier (by ensuring scarring and wound healing), (ii) the local secretion of anti- and pro-inflammatory cytokines (thus modulating both metabolic and immune components of metabolic homeostasis), (iii) immunosurveillance by AT-resident macrophages, and (iv) the direct production of antimicrobial peptides (such as cathelicidins) by adipocytes. Locally, both innate and adaptive immune cells contribute to anti-infectious responses. The systemic contribution of AT to immune responses against infection is mainly driven by the secretion of anti- or pro-inflammatory cytokines that modulate immune responses, regardless of the target antigen (i.e., pathogen, tumor, or self-antigens). However, the recent discovery of the systemic role played by resident memory T cells in non-lymphoid tissue means that one must consider a plethora of new ways in which immune cells in AT may contribute to memory immune response. The accumulation of memory T cells in AT close to interface and lymphoid structures may be a decisive factor in the development of a memory response against a secondary infection by a previously encountered pathogen.

Our understanding of AT’s contribution to anti-infectious immune responses has changed over time (Figure 4). AT was initially considered to be an inert mechanical barrier – a buffer site that protects against mechanical trauma and thus protects organs from breach and subsequent infections. In the early 2000s, innate immune cells were found in AT (Zeyda and Stulnig, 2007; Schaffler and Scholmerich, 2010). In particular, the AT-resident macrophage fraction was found to contribute to strong pro-inflammatory responses with both local and paracrine effects (Hotamisligil, 2006; Ouchi et al., 2011; Jackson et al., 2017). Other immune cells (CD8 T cells and NK cells) have been identified more recently, although the cells’ local effects have mainly been studied with regard to metabolic homeostasis. Our knowledge of AT’s anti-infectious potential has also benefited from research showing that adipocytes *per se* exert antimicrobial responses (Nippe et al., 2011; Alcorn and Kolls, 2015). Lastly, the discovery of memory T cell accumulation in AT as a means of providing efficient secondary responses against pathogens constituted a major breakthrough; it became clear that AT is an immune partner in both local and systemic immune responses (Han et al., 2017; Figure 5). However, AT is a reservoir for pathogens in various infectious contexts (Nishimura et al., 2000; Neyrolles et al., 2006; Desruisseaux et al., 2007; Dhurandhar, 2011; Nagajyothi et al., 2012; Couturier et al., 2015, 2016; Damouche et al., 2015; Trindade et al., 2016; Beigier-Bompadre et al., 2017; Tanowitz et al., 2017; Couturier and Lewis, 2018; Contreras et al., 2019; Table 2). The detection of various bacterial DNAs in AT also constitutes an argument in favor of the tissue-specific microbiota hypothesis (Burcelin et al., 2013). The unexpected coexistence of immune cells and pathogens within AT is intriguing, and warrants further evaluation with regard to anti-infectious immune responses. Here are some points to discuss for considering a role in immunity of AT besides its close proximity to sites of entry for infective agents.

**TABLE 2:** Metabolic and anti-infectious activity of immune cells in adipose tissue.

**Cell subsets**	**Modulation associated with obesity**	**Metabolic activity**	**Anti-infectious activity**	**References**
Macrophages	↗ Shift from M2 to M1	Subset-dependent M2: Phagocytosis of dying adipocytes Lipid buffering Limiting inflammation M1: TNF-α production inducing insulin resistance in adipocytes Favoring angiogenesis	M1: TNF-α, MIP-1α producer	Weisberg et al., 2003; Lumeng et al., 2007; Catrysse and van Loo, 2018; Ivanov et al., 2018; Lumeng et al., 2007; Morris et al., 2013; Suganami et al., 2007; Pang et al., 2008
CD4 T cells	Shift from Th2 to Th1	Subset dependent Th2: maintaining glucose homeostasis Th1: promoting inflammation		
CD8 T cells	↗	Rare at steady state, may contribute to the elimination of compromised adipocyte Early promoter of obesity-induced inflammation	Increased proportion in various contexts of AT infection Contributing to anti-infectious memory responses	Rausch et al., 2008; Nishimura et al., 2009b; Han et al., 2017
NKT cells	↘	Regulator of adipose tissue function Th2 cell type cytokine production		
Treg	↘	Regulator of adipose tissue function	Control of adipocyte function Lipid uptake and metabolism	Feuerer et al., 2009; Cipolletta et al., 2012, 2015; Kolodin et al., 2015; Laparra et al., 2019
NK cells	↗ Stimulated phenotype	Subset dependent Early sensor of adipose stress – At steady state, eliminating macrophage – When activated, triggering M1 macrophage accumulation and adipose fibrosis		
ILC2	↘	Regulator of adipose tissue function Favoring beiging (Met-Enk, IL4) Favoring Type 2 innate immunity	Promoting B cell response, IgM production	Moro et al., 2010; Brestoff et al., 2015; Lee et al., 2015

### Anti-infectious Immunity in Adipose Tissue

We will distinguish the local and systemic immune responses developed by AT. Indeed, the systemic activity of AT was initially related to the endocrine cytokine production but need to be reconsidered with the contribution of AT CD8 T cells to anti-infectious secondary responses.

#### Local Immunity

##### Soluble factors and adipokines

Adipose tissue produces a broad range of molecules referred to as adipokines. Although some adipokines (such as adiponectin) are produced solely by adipocytes, most (including cytokines and chemokines) are produced by AT-resident immune cells (Fain et al., 2004) [notably macrophages (Ouchi et al., 2011)]. Although the endocrine activity of AT is pivotal, the local effect of the adipokines is non-negligible. Some adipokine such as TNF-α are not secreted into the circulation and exert local activity (Mohamed-Ali et al., 1997; Braune et al., 2017). Low-grade local inflammation is essential for remodeling healthy AT and healing wounds efficiently (Wernstedt Asterholm et al., 2014), whereas higher-grade inflammation helps to modulate immune responses against pathogens. Leptin (whose primary role is to regulate metabolism) is probably a prototypic example of the close link between the metabolism and the immune system (Matarese and La Cava, 2004). Leptin promotes T-cell proliferation, T-cell activation, and T-helper cell polarization *in vitro*. As a whole, AT inflammation is described in various models of infection (Nagajyothi et al., 2012; Contreras et al., 2019), underscoring that anti-infectious responses develop in AT.

##### The stromal vascular fraction

Little information is available on the anti-infective properties of TA, as studies have mainly evaluated their contribution to metabolic functions (Table 3). Healthy AT is associated to an anti-inflammatory environment, that may theoretically limit the anti-infectious potential of AT. Among type 2 immune subsets present in AT, Tregs and ILC2 are central partners of AT regulation, providing anti-inflammatory signals by different pathways. One specific feature of non-obese VAT is the high proportion of CD4 Tregs, which account for up to 50–70% of CD4 T cells in VAT in 25-week-old male mice (Feuerer et al., 2009). Interestingly, the Tregs in AT express the transcription factor PPAR-γ that regulates fatty acid storage and glucose metabolism (Cipolletta et al., 2012). Adipocytes express PPAR-γ, the most notable function of which is the regulation of adipogenesis. The presence of a PPAR-γ signature in AT Tregs has been considered as a marker of these cells’ functional plasticity; the Tregs express the very same transcription factor produced by the cells on which they exert a modulatory effect (Cipolletta et al., 2011; Cipolletta, 2014). In contrast to peripheral Tregs, rodent adipose Tregs also express transcripts coding for several proteins involved in lipid metabolism (Pcyt1a and Dgat1), and are capable of lipid uptake (Cipolletta et al., 2012, 2015; Kolodin et al., 2015). Unfortunately, the high proportion of Tregs in VAT has not been confirmed in humans or non-human primates (NHPs) (Damouche et al., 2017). Given that the immune cell populations in AT are strongly modulated by inflammatory and/or metabolic signals and microbiota, the lower proportion of Tregs in human VAT (relative to murine VAT) might reflect differences in the metabolic and microbial challenges faced by humans vs. rodents. However, subsequent analyses of Treg counts in various mouse strains revealed that the high observed values were restricted to the VAT of male C57Bl/6 mice of a certain age (Laparra et al., 2019). Whereas the proportion of AT Tregs is much lower in obese rodents than in lean rodents [which presumably influences ongoing, obesity-associated inflammation (Cipolletta et al., 2012)], there is less agreement about whether this is true in humans. Although this disparity may reflect the fact that Foxp3 staining is less specific for human Tregs than for mouse Tregs (which could explain the preserved expression of Foxp3+ cells in obesity due to the accumulation of effector cells) and/or different metabolic and infectious histories in humans and mice, it may also reflect a model-specific difference in the contribution of the Treg subset in AT. Therefore, the potential immunosuppressive role of Tregs (i.e., limiting the development of an efficient anti-infectious immune responses) may not be as relevant in humans as first thought. The role of AT Tregs in the maintenance of human metabolic homeostasis is also subject to debate.

**TABLE 3:** Microbes detected in adipose tissue.

**Pathogens**	**Species**	**Infected cell subset**	**Impact on AT biology**	**References**
Human adenovirus	m			Dhurandhar, 2011
*Trypanosoma gondii*	m			Machado et al., 2012; Nagajyothi et al., 2012; Trindade et al., 2016; Tanowitz et al., 2017
*Plasmodium berghei*	m			Franke-Fayard et al., 2010
*Plasmodium falciparum*	Hu			Seydel et al., 2006
*Leishmania* parasites	m	ASCs		Allahverdiyev et al., 2011
*Nippostrongylus brasiliensis*			↗ Eosinophils	Wu et al., 2011; Hussaarts et al., 2015
*Mycobacterium tuberculosis*	m, Hu	Adipocytes SVF cells	↗ CD8 T cells and NK cells, ↗ IFN-g production	Neyrolles et al., 2006; Beigier-Bompadre et al., 2017
Microbiota/bacterial DNA				Burcelin et al., 2013
*Chlamydia pneumoniae*	*In vitro*			Bouwman et al., 2008
*Rickettsia prowazekii*				Bechah et al., 2010
*Staphylococcus aureus*	*In vitro*	Adipocytes		Hanses et al., 2011
Influenza virus	Hu			Nishimura et al., 2000
Respiratory syncytial virus	*In vitro*			Bouwman et al., 2008
HIV	NHP, Hu		↗ CD8 T cells	Couturier et al., 2015; Damouche et al., 2015
Cytomegalovirus	m	Adipocytes Endothelial cells	Metabolic changes ↗ CD8 T cells	Price et al., 1990; Sacher et al., 2011; Contreras et al., 2019

*Species in which the microbes have been identified are indicated (m, mouse; Hu, human; NHP, non-human primate). When available, the cell subset targeted by the microbes and the impact on the immune compartment is indicated.*

Group 2 innate lymphoid cells are also a crucial subset involved in the regulation of AT function (Molofsky et al., 2013). First described in FALCs in the mesentery, their identification has been extended to the whole AT. ILC2s contribute to the conversion of white to beige AT by directly impacting adipocyte progenitors (Brestoff et al., 2015) and thus preventing excessive energy storage. They also act indirectly by favoring eosinophils and M2 macrophages maintenance (Lee et al., 2015). Interestingly, ILC2 also provide help to B cells and contribute to IgA production, thus potentially contributing to immune responses.

Importantly, these cells are subjected and strongly regulated by the metabolic context. A detailed analysis of anti-infective immune responses would require the study of immune responses in both healthy and obese AT. Increases in macrophages, Th1 CD4 T cells, and CD8 T cells are described in most metabolic (McLaughlin et al., 2014; Liu and Nikolajczyk, 2019) and infectious episode affecting AT (Nagajyothi et al., 2012; Beigier-Bompadre et al., 2017), as well as inductive lymphoid structures (Rangel-Moreno et al., 2009; Bénézech et al., 2015, Bénézech and Jackson-Jones, 2019). These similarities question the potential synergy between obesity and anti-infectious immune responses. As in any non-lymphoid sites, the recruitment of innate and adaptive immune cell defines AT anti-infectious local responses but the AT local properties may impact their anti-infectious potential.

#### Adipocytes: The Immune Side of the Metabolic Cells

A number of studies have found that adipocyte lineage cells possess innate immune activity. Early-stage adipocyte precursors (such as preadipocytes) are known to have certain macrophage-like properties (Cousin et al., 1999; Charrière et al., 2003; Saillan-Barreau et al., 2003). The adipocytes’ direct contribution to the immune response during infection is also suggested by the cell surface expression of Toll-like receptors (which sense conserved microbial motifs) on adipocytes. Stimulation of these receptors induces the production of pro-inflammatory cytokines (Lin et al., 2000). The expression of MHC class II molecules (crucial for CD4 T cell survival and activation) at the surface of adipocytes has also been described (Meijer et al., 2011; Deng et al., 2013). Deng et al. (2013) demonstrated that MHC class II expression on adipocytes was essential for the development of obesity-induced adipose inflammation. Adipocytes may act as antigen-presenting cells for T lymphocytes, although they do not enable full activation (Deng et al., 2013; Hruskova and Biswas, 2013). Zhang et al. (2015) recent report is even more striking; the researchers demonstrated that dermal adipocytes exerted direct antimicrobial activity against *Staphylococcus aureus* by producing cathelicidins – thus unequivocally confirming AT as a crucial component of innate responses to infection (Alcorn and Kolls, 2015; Kugelberg, 2015; Zhang et al., 2015).

#### Systemic Immunity

##### The endocrine activity of adipose tissue

Once the AT’s endocrine activity had been demonstrated, the impact of various adipokines (including hormones, cytokines, and chemokines produced in AT) on the immune system was widely documented. Leptin, IL-1, IL-6, IL-8, interferon gamma (IFN-γ), transforming growth factor beta (TGF-β), and chemokines (such as monocyte chemotactic protein-1 and macrophage inflammatory protein-1) produced by AT interfere with the remote immune system. T cells and antigen-presenting cells are influenced by leptin, IL-6, and insulin. Conversely, in a context of calorie restriction and starvation, levels of pro-inflammatory adipokines fall and those of anti-inflammatory adipokines rise, which contributes to immunosuppression. Although not specific to the immune responses directed against pathogens, AT, by the secretion of various adipokines modulate immune responses.

Various murine models indicated that obesity (i.e., excessive adiposity) increases susceptibility to infection. Obese mice are more susceptible to bacterial infections due to greater microbial load and/or impaired clearance (*Klebsiella*, *Mycobacterium tuberculosis*, *Staphylococcus*) (Mancuso et al., 2002; Ikejima et al., 2005; Wieland et al., 2005), impaired resistance to listeria monocytogenes infection (Ikejima et al., 2005). However, most of these results were obtained using leptin-deficient *ob/ob* mice which are resistant to a variety of experimental models of inflammation/autoimmunity (La Cava et al., 2004; Matarese and La Cava, 2004), and therefore cannot be considered as a model which fully recapitulates the pathophysiology of obesity. Overall, obesity appears to be associated with higher susceptibility to infection (Karlsson and Beck, 2010; Huttunen and Syrjänen, 2013) even if the mechanisms involved remain unclear.

##### Adipose-tissue-resident memory T cells

It has been consistently observed that CD8 T cells accumulated in AT when the latter is exposed to metabolic or infectious insults. Whereas the clonal specificity of these cells is not clear in a metabolic context, pathogen-specific CD8 T cells have been found in AT. Although AT exhibits some very specific features, it is tempting to speculate that AT-Trm cells behave in much the same way as other memory T cells resident in non-lymphoid tissues. The literature on the biology of Trm cells is extensive (Gebhardt et al., 2009; Mueller et al., 2013; Sathaliyawala et al., 2013). Beura et al. (2018) recently described how CD8 Trm cells (but not CD4 Trm cells) (Beura et al., 2019) migrate from non-lymphoid tissue to nearby lymphoid sites. Resident memory T cells may thus support a secondary immune response produced at lymphoid sites during rechallenge. If this phenomenon occurs in AT, it would highlight the tissue as a major partner in memory T cell responses. Interestingly, Han et al.’s (2017) important study demonstrated that CD8 T cells from AT can indeed produce secondary immune responses. By grafting AT collected from animals infected with *Toxoplasma gondii*, the researchers demonstrate that CD8 T cells in the graft protected the naïve recipients from what would normally be a lethal challenge with the same pathogens (Han et al., 2017).

The phenotypic characterization of Trm cells is ongoing (Walsh et al., 2019), and it is not yet clear whether or not the memory CD8 T cells found in AT are indeed resident. In non-lymphoid sites, the expression of surface markers like CD103 and CD69 is usually considered to be a marker of residency. CD69 is strongly expressed by CD4 and CD8 T cells in AT (Couturier et al., 2015; Damouche et al., 2017). However, given that CD69 is also an activation marker, the expression of CD69 on memory T cells in AT has been linked to activation as well as residency. Importantly, the two notions are not necessarily exclusive; the tissue-specific expression of CD69 may reflect the low-grade stimulation and activation of T cells, which would favor their persistence. The results of parabiosis experiments tend to support the existence of Trm cells in AT (Han et al., 2017).

In conclusion, AT’s potential role in innate and adaptive immune responses to infection is based on the tissue’s immune cell content, pro-inflammatory potential, and proximity to sites with immune activity. Lastly, the recent realization that Trm T cells contribute to memory responses at lymphoid sites drastically broadens the anti-infectious potential of AT; this tissue might support local and systemic cell-based anti-infectious responses (Figure 4).

### Adipose Tissue as a Pathogen Reservoir, and Pathogen-Induced Perturbations

Our group and others have demonstrated that AT is a reservoir for HIV (Couturier et al., 2015, 2016, 2018; Damouche et al., 2015; Hsu et al., 2017). Interestingly, a number of pathogens persist in AT – prompting the question of whether HIV’s persistence in AT relies on a specific targeting mechanism or reflects the AT’s broader, intrinsic ability to store pathogens. Although the marked accumulation of pathogens in AT is striking, the cell subsets that define the cellular reservoir differ (Table 2) – suggesting the presence of pathogen-specific mechanisms.

Pathogens frequently reported in HIV co-infections [such as *M. tuberculosis* (Beigier-Bompadre et al., 2017) and *Leishmania* parasites (Allahverdiyev et al., 2011)] have been detected in AT. Interestingly, the cytomegalovirus (CMV, a herpes virus that infects most people worldwide) has been also detected in AT (Contreras et al., 2019). The impact of CMV infection on aging has been widely documented (Solana et al., 2012; Wertheimer et al., 2014), and HIV infection has also been linked to accelerated aging (Appay et al., 2007, 2011). In a context of HIV, interaction with other pathogens present in AT might accentuate the specific effects of the viral infection. Conversely, HIV may accentuate a preexisting chronic tuberculosis (TB) or *Leishmania* infection locally.

#### *Mycobacterium tuberculosis* in Adipose Tissue

Tuberculosis is one of the most common co-infections in people living with HIV. In non-treated patients with latent TB, the immunosuppression associated with HIV infection is particularly harmful because it may trigger the development of an active TB infection. Treating a TB and HIV co-infection is clinically challenging; drug interference and toxicity prevents the respective treatments from being combined, leading clinicians to favor one treatment over the other (Djimeu and Heard, 2019). It has been shown that TB-associated immune reconstitution inflammatory syndrome can develop when ART is initiated (Worodria et al., 2011; Abay et al., 2015; Haridas et al., 2015; Boulougoura and Sereti, 2016). Importantly, ART-controlled HIV infection appears to interfere with TB infection (Worodria et al., 2018; Holmberg et al., 2019).

*Mycobacterium tuberculosis* can be detected in both SVF cells (macrophages) and adipocytes. Furthermore, the transfer of AT lysates from infected mice to naïve recipients can induce a TB infection. The local persistence of *M. tuberculosis* is associated with changes in the AT’s immune compartment, with infiltration of CD8 T cells and NK cells and the production of IFN-γ (Neyrolles et al., 2006; Beigier-Bompadre et al., 2017).

#### Cytomegalovirus in Adipose Tissue

As mentioned above, CMV is a herpesvirus that infects most of the world’s population. The long-term impact of chronic CMV persistence has been extensively studied. Infection by CMV is associated with immune senescence, and is a prognostic component of the immune risk profile in elderly adults. CMV and HIV infections are interlinked; accentuated CMV-specific memory T-cell responses associated with an immune risk phenotype constitute a prominent immunologic feature of accelerated aging in HIV infection (Barrett et al., 2012). Conversely, the efficacy of ART treatment (restoration of the CD4 T cell count) depends on the patient’s CMV status (Appay et al., 2011; Sauce et al., 2011). The reason for the connection between HIV and CMV is subject to debate but may be related to the viruses’ apparent colocalization in AT.

Cytomegalovirus infection of AT is also associated with the infiltration and/or local expansion of CD8 T cells, the development of pro-inflammatory responses, and metabolic alterations (Contreras et al., 2019). Interestingly, signs of aging have also been associated with HIV infection; it remains to be established whether a synergistic effect of the various local infections may aggravate alterations in AT’s metabolic functions.

### Human Immunodeficiency Virus in Adipose Tissue

The link between AT and HIV infection has long been known. People infected with HIV suffer from metabolic alterations (including dyslipidemia, insulin resistance, and lipodystrophy) that were initially considered to be adverse reactions to the first classes of antiretroviral drugs (ARVs) to be developed [i.e., nucleoside reverse transcriptase inhibitors (NRTIs) and protease inhibitors (PIs)] (Samaras et al., 2007; Falutz, 2011). However, the presence of metabolic alterations in treatment-naïve HIV-infected people suggests that the virus has a direct impact. Accordingly, researchers sought to determine whether HIV infects adipocytes, since infection might explain the broad disruption of fat biology (either alone or by accentuating the toxicity of ARV drugs). These studies were performed in the early 2000s. Although adipocytes (i) express the co-receptors required for HIV entry (Hazan et al., 2002), and (ii) can be infected *in vitro* (Maurin et al., 2005), HIV has never been directly detected in these cells (Dupin et al., 2002; Munier et al., 2003). The spread of viral proteins has also been studied, and viral proteins such as Vpr have indeed been found in AT from HIV-infected patients. The viral proteins’ contribution to adipose dysfunction has been studied (Muthumani et al., 2005; Shrivastav et al., 2008; Agarwal et al., 2013). In the meantime, however, studies of AT alterations in obesity revealed the high proportion of immune cells (and notably CD4 T cells and macrophages, HIV’s two main targets) in AT. When considering the SVF in particular, a different picture emerged: HIV (or SIV, in macaque models) was detected (Couturier et al., 2015, 2016; Damouche et al., 2015) in both viremic and controlled ART-treated patients. The virus was also found in the macrophage fraction in the viremic macaque model but not in samples from ART-treated HIV-infected patients. The low macrophage count in these patients precluded any conclusions as to the respective levels of macrophage infection during the viremic and aviremic phases.

Several research groups have confirmed that HIV infects CD4 T cells in AT (Damouche et al., 2015; Couturier et al., 2016) and that this virus is replication-competent (Damouche et al., 2015; Couturier et al., 2018; Table 4). However, these and other findings raise a number of questions. Firstly, there are few data on the time course of AT infection and the mechanism that underlies HIV infection and persistence in AT cells. It appears that AT cells are infected quite rapidly (within 4 weeks: personal data). Secondly, the CD4 T cell fractions in SAT and VAT exhibit similar level of HIV DNA during the chronic phase of the infection – suggesting that the common features of SAT and VAT (rather than the specific properties of each type of AT) favor viral infection and/or persistence. However, the specific factors that allow viral persistence in AT have yet to be defined. Thirdly, viral persistence of HIV in many other AT depots (e.g., perivascular, dermal, and BM depots) has not been evaluated. Fourthly, there are few data on the impact of obesity on HIV infections and the establishment of the HIV reservoir in AT. From a technical standpoint, studies of most of these questions are limited by the small number of cells recovered from AT samples.

**TABLE 4:** Studies of the presence of HIV (or the simian form, SIV/SHIV) in adipose tissue in humans and simians, indicating the detection strategies and pathogens.

**Year**	**Species**	**Detection of viral nucleic acids in adipose tissue**	**References**
2002	Hu	No HIV DNA in adipose tissue lysate	Dupin et al., 2002
2015	Hu	SHIV DNA in SVF cells	Couturier et al., 2015
	Cyno Ms, Hu	SIV DNA and RNA in SVF cells, SVF CD4 T cells and SVF macrophages HIV DNA and RNA in SVF cells, SVF CD4 T cells *in vitro* reactivation assay	Damouche et al., 2015
2016	Rh Ms	SHIV DNA in SVF cells	Couturier et al., 2016
2017	Rh Ms	SHIV RNA in SVF and SVF CD4 T cells at week 2	Hsu et al., 2017
2018	Hu	HIV DNA in SVF CD4 T cells	Koethe et al., 2017
2018	Hu	*In vitro* reactivation assay	Couturier et al., 2018

*Cyno Ms, cynomolgus macaque; Rh Ms, rhesus macaques.*

## Mechanisms That May Favor the Persistence of HIV in Adipose Tissue

Very little information is available on the mechanisms that may favor HIV persistence but various hypotheses may be formulated (Table 5).

**TABLE 5:** Mechanisms that may favor the persistence of HIV in adipose tissue.

**Parameters potentially favoring viral persistence in adipose tissue**		**References**
**Immunological parameters**	**Mechanisms/observations**	
AT as an Immunosuppressive environment		Wan et al., 2008; Khairoun and Korevaar, 2013; Yousefi et al., 2016
ASC	Limiting T cell proliferation	Khairoun and Korevaar, 2013
	Favoring Treg (generation and accumulation)	Yousefi et al., 2016
Th2-like cells and cytokines		
**Limited efficiency of Ag presenting cells**		
Distinct local distribution of CD4 and CD8 T cells		Damouche et al., 2015
Defective T cell responses		Wronska and Kmiec, 2012
Exhaustion	High percentage of PD-1 expressing cells	Shirakawa et al., 2016; Damouche et al., 2017
Senescence	High percentage of CD57 expressing cells	Koethe et al., 2017
** Metabolic reprogramming**		
Resident memory T cell		Pan et al., 2017
CD36 expression on AT T cell		Couturier et al., 2019
**Metabolic parameters**		
Interaction between adipocyte and immune cells The consequences of a lipid-rich environment		Mazzon and Mercer, 2014
Oxidative stress		Malmberg et al., 2001; Ivanov et al., 2016
Hypoxia in adipose tissue		Tao et al., 2015
Insulin sensitivity		Tsai et al., 2018
**Viral and pharmacological parameters**		
HIV viral proteins		Boya et al., 2004; Debaisieux et al., 2012; Clark et al., 2017; Jacob et al., 2017
Adipose tissue remodeling and fibrosis		Sun et al., 2013; Vila et al., 2014; Marcelin et al., 2017
Antiretroviral diffusion (ARV)		Dupin et al., 2002; Couturier et al., 2018

### Immunologic Parameters

Both of HIV’s main targets (CD4 T cells and macrophages) are present in AT. Both cell types are predominantly in a resting phase, which may favor the latency of HIV. The proximity to a major site of viral replication (such as the gut mucosae) may also contribute to the direct infection of AT. However, SAT or VAT do not differ significantly with regard to the level of HIV DNA in CD4 T cells (Damouche et al., 2015) – suggesting that environmental factors (e.g., low-grade inflammation, local microbial inflammation, and the viral load) do not drastically affect the level of infection of the CD4 T cell compartment.

#### Adipose Tissue as an Immunosuppressive Environment

One possible mechanism for HIV persistence in AT would involve local immunosuppression and thus failure to eliminate HIV-infected cells. An obvious candidate for this immunosuppression is the Foxp3+ CD4 Treg subset. We compared the distribution of the various cell subtypes associated with immunosuppression in the AT of mice, NHPs, and macaques (Laparra et al., 2019). In AT from adult male humans and NHPs, we found a low proportion of Tregs, MSCs, and other subsets with immunosuppressive functions – thus ruling out a major role of Treg inhibition in the persistence of HIV in AT. Importantly, we observed a small but statistically significant increase in the proportion of Foxp3+ Treg cells in SAT from ART-treated HIV-infected patients, relative to SAT from non-infected control subjects (Damouche et al., 2017) – suggesting that the changes induced by HIV infection in AT may also contribute to viral persistence. Adipocytes may represent the main Ag-presenting cells subset in AT (representing >50% of AT cell), but may lack costimulatory signals that are required for efficient presentation and immune cell activation (Meijer et al., 2011; Deng et al., 2013). However, data demonstrating that adipocytes ensure efficient T cell stimulation (Xiao et al., 2016) and the presence of DCs in AT emphasize the need to decipher the capacity for Ag presentation by AT.

#### T Cell Exhaustion and Senescence

Another intrinsic property of AT is the high surface expression of PD-1 (an exhaustion marker) on both CD4 and CD8 T cells. Although it is not known whether or not cells in AT cells express PD-L1 or PD-L2, the presence of PD-1 indicates high susceptibility to exhaustion. The difference in PD-1 expression between AT-resident T cells and circulating T cells is observed on both CD4 and CD8 T cell subsets, but reflect two distinct processes. Indeed, the high PD-1 expression on CD4 T cells in AT is due to the higher proportion of memory CD4 T cells which intrinsically express PD-1, and not to a higher expression level in CD4 T cells in AT compared with blood. At the opposite, memory CD8 T cells collected from AT or blood differ with regard to PD-1 expression – suggesting that a phenotypic change within CD8 T cell subsets is associated with the cells’ persistence in the AT environment. Regardless of the mechanisms, AT resident T cells exhibit high proportion of PD-1 expressing cells.

Similarly, AT resident CD4 and CD8 T cells also expressed high proportion of CD57 expressing cells (Koethe et al., 2017; Wanjalla et al., 2019). CD57 expression is associated with both terminal differentiation and replicative senescence, providing mixed signals regarding their functional potential.

#### CD4 and CD8 T Cells Are Not Colocalized in AT

We have studied the distribution of CD4 and CD8 T cells in AT from ART patients (Damouche et al., 2015). In both HIV-infected and non-infected donors, we observed that CD4 and CD8 T cells were generally not colocalized. This provides a strong rationale for the persistence of HIV-infected CD4 T cells, which could not be reached by CD8 T cells. This observation obviously parallels the situation described in LNs, whereas follicular helper CD4 T cells are also out of reach of CD8 T cells and thus persist in the germinal center. It remains to be determined whether or not metabolic alterations that disturb the AT architecture can modify this non-colocalization and thus induce clusters with direct contact between CD4 and CD8 T cells. The absence of CD4–CD8 T cell contact may have a direct impact on the persistence of HIV-infected CD4 T cells, and might also interfere with the effective differentiation of memory cells. Indeed, CD4 T cell help is a crucial factor in the differentiation and maintenance of CD8 T cells.

#### HIV Viral Proteins Affecting Immune Cells

The immune compartment can be modulated not only by AT’s intrinsic properties but also by HIV infection. It has been shown that the HIV proteins Tat, Nef, gp120, and Vpr can modulate immune cells, notably by inducing the apoptosis of non-infected CD4 T cells as a bystander effect (Boya et al., 2004). Nef binds to CXCR4 and modulates T cell activation (Jacob et al., 2017), and so may also be involved in HIV persistence. Tat induces apoptosis in non-infected CD8 and CD4 T cells, and alters the metabolic, morphologic, and biochemical properties of CD4 T cells (Debaisieux et al., 2012; Faller et al., 2014; Clark et al., 2017). Vpr lengthens the lifespan of HIV-infected macrophages by modulating glutamate metabolism in the mitochondria (Datta et al., 2016).

#### Metabolic Reprogramming of Immune Cells in AT

Recent research has characterized the metabolic requirements of functionally effective immune cells. Metabolic reprogramming of immune cells is a crucial aspect related to their differentiation status. Resting, memory, and regulatory T cells have different metabolic requirements: activation of T cells is accompanied by a switch from oxidative metabolism to intensified glucose metabolism *via* aerobic glycolysis. Tregs have reduced ability to activate the PI3K/Akt pathway (Palmer et al., 2016a, b). Metabolic reprograming of immune cells is also described in non-lymphoid tissues: Trm cell survival depends on exogenous lipid uptake (Pan et al., 2017). T lymphocytes in AT express high levels of CD36, a scavenger receptor for lipid uptake (Couturier et al., 2019).

Viruses are obligate parasites that are completely reliant on host cell metabolism. They manipulate cellular metabolism including glycolysis, fatty acid synthesis, and glutaminolysis to create an intracellular metabolic niche, which supports virion production and promotes survival of infected cells. Increased Glut1 expression on CD4 T cells in culture increases cellular permissivity to HIV-1 infection, while suppression of glucose metabolism by PI3K inhibitors inhibits infection (Loisel-Meyer et al., 2012). Lastly, Saez-Cirion et al.’s recent study (Valle-Casuso et al., 2019) showed that HIV specifically targets highly metabolically active CD4 T cells (i.e., enabling the virus to multiply more rapidly). HIV-1 selectively infects CD4 T cells with high levels of oxidative phosphorylation and glycolysis, independently of their activation phenotype. In a similar way to carcinogenesis, it is also now admitted the existence of a metabolic reprogramming upon HIV infection, related to modifications in glucose metabolism (Ahmed et al., 2018).

In this respect, AT provides very specific metabolic cues whose effects on the local immune responses (and the virus cycle) requires further investigation. Whether AT immune cells in AT may be sensible to these HIV-induced reprogramming or favor viral latency remain to be evaluated.

### Metabolic Parameters

Initially, the term “immunometabolism” referred to the two-way interaction between the metabolism and the immune system. This entity has now been subdivided into “tissue immunometabolism” (the impact of immune cells on both local and systemic metabolic homeostasis, and vice versa) (Matarese and La Cava, 2004; Ferrante, 2013) and “cellular immunometabolism” (the intracellular metabolic changes associated with immune cell responses and survival) (Wang and Wu, 2018). Importantly, viruses need to subvert the cell’s metabolism to develop, and so virus–metabolism interactions are now also considered. With regard to the specific metabolic properties of AT, various factors (including the ECM in AT) may directly or indirectly favor viral infection and/or persistence.

#### The Consequences of a Lipid-Rich Environment

Lipids fulfill three general functions: (i) energy storage, principally as TG and steryl esters in lipid droplets, (ii) structural components of the cell (plasma and organelle membranes, and membrane component of budding and intracellular trafficking), and (iii) first and second messengers in signal transduction (van Meer et al., 2008). With regard to the latter point, several studies have found that the type and quantity of fatty acids and lipid mediators can influence the immune system in a potent manner (Pal et al., 2012). Saturated fatty acids are able to activate Toll-like receptors on adipocytes *via* endogenous ligand fetuin-A (Pal et al., 2012), and are involved in inflammation. Unsaturated fatty acids can be oxidized to generate potent pro-inflammatory or pro-resolving lipid mediators. High concentrations of fatty acids are toxic for T cells, whereas non-toxic concentrations can induce proliferation and cytokine production. This aspect of the interaction between immune cells and metabolic signals is probably the best documented (Ferrante, 2013), although the direct influence of the AT environment on immune responses during infection requires further investigation. Furthermore, studies of the interactions between viruses and host cells have also revealed the role played by structural lipids during each step in the viral lifecycle (binding, internalization, fusion, genome uncoating, replication, particle assembly, and budding) (Mazzon and Mercer, 2014). In the early stages of infection, viruses subvert cellular lipids and use lipid-based signaling mechanisms for entry and trafficking. Once an infection has developed and viral genes have been expressed, lipid synthesis is extensively reprogrammed and the lipid distribution is remodeled to promote viral replication, assembly, and egress. It is known that HIV actively modulates lipid rafts by increasing the synthesis and trafficking of cholesterol to these sites. Furthermore, soluble HIV-1 proteins can enter cells through lipid rafts (Mañes et al., 2003). These observations suggest that the lipid-rich environment in AT may have a direct impact on plasma membrane composition and plasticity (notably the lipid rafts). In the context of tumors, an increase in circulating cholesterol levels is associated with an increase in the cholesterol content of lipid rafts; in turn, this alters raft-mediated downstream signaling and promotes tumor growth (Mollinedo and Gajate, 2015). It is not clear whether the structural composition and plasticity of plasma and organelle membranes of the main cellular targets of HIV differ in AT (relative to other tissues), although these factors might contribute to preferential infection by HIV and facilitated viral latency. Unfortunately, very little is known about the putative specific influence of the AT’s lipid-rich environment on the lifecycle of HIV.

#### Oxidative Stress

Adipose tissue produces reactive oxygen species (ROS) under various circumstances, and especially in a context of obesity. Both chronic and acute exposure to oxidative stress affects adipocyte and T cells (Malmberg et al., 2001), and also appears to impact HIV’s lifecycle (Ivanov et al., 2016). In fact, HIV infection triggers pronounced oxidative stress, which subsequently enhances viral transcription and creates a positive feedback loop. The Vpr protein induces hypoxia inducible factor 1 (HIF-1) and then the ROS-dependent activation of HIV’s long terminal repeat. Pro-inflammatory cytokines (such as TNF-α) also enhance viral transcription. Importantly, glutathione treatment of chronically infected cells during the later stages of the HIV life cycle leads to the abrogation of virion budding and release (Beura et al., 2018), which potentially favors viral latency. The direct impact of oxidative stress in the AT on the HIV and HIV-infected cells has yet to be evaluated in detail. Given the known functional impact of oxidative stress on the immune cells and adipocytes in AT, ROS production may also affect HIV’s infectivity and persistence.

#### Hypoxia in Adipose Tissue

As adipocytes expand, the interstitial oxygen tension falls. The subsequent activation of HIF-1 inhibits pre-adipocyte differentiation and initiates AT fibrosis. HIF-1 is also expressed by T cells and other immune cells, and is a potent regulator of T cell survival, activation, and differentiation (McNamee et al., 2013; Tao et al., 2015). It is known that HIF-1 drives pro-inflammatory Th17 cell function and longevity. Lastly, low oxygen levels inhibit the replication of HIV-1 and its reactivation from latent reservoirs (Charles et al., 2009; Loisel-Meyer et al., 2012; Nekhai et al., 2013). As previously described for the lipid-rich environment and oxidative stress, the intrinsic impact of the AT environment must be taken into account.

#### Insulin Sensitivity

Insulin has a critical role in maintaining the homeostasis of energy metabolism, and coordinates the storage and utilization of fuel molecules in AT and in other insulin-sensitive peripheral tissues. Recent research has highlighted the two-way link between insulin and immune cells (Dalmas et al., 2017; Tsai et al., 2018), and has confirmed that metabolic parameters can indirectly influence immune responses. However, this two-way link has not been observed in AT, and a direct relationship between insulin sensitivity and the HIV life cycle has not been demonstrated at the cellular level.

#### Adipose Tissue Remodeling and Fibrosis

In addition to intrinsic metabolic–immune interactions in healthy AT, metabolic and immune remodeling can also be induced by HIV infection and ART treatment. Remodeling of the ECM is a key event in HIV infection, and HIV infection has a specific role in AT fibrosis. Macrophages and ASCs are major players in the onset of AT fibrosis (Vila et al., 2014; Marcelin et al., 2017), notably by the production of TGF-β. It has been reported that SIV-infected macaques upregulated TGF-β expression in the SAT and VAT (Gorwood et al., 2019). The HIV-1 proteins Tat and Nef are secreted by infected cells and then induce a profibrotic phenotype in ASCs. The increase in collagen production and TGF-β secretion associated with HIV infection may be involved in AT fibrosis. The TGF-β produced by profibrotic adipose-derived MSCs might also have an impact on the Tregs in AT, although this topic requires further investigation. The persistence of an inflammatory stimulus and/or hypoxia in AT may be responsible for the excessive synthesis of ECM components and the subsequent interstitial deposition of fibrotic material (Sun et al., 2013). This collagen deposition is accompanied by the infiltration of macrophages and other immune cells (Spencer et al., 2010). Although fibrosis directly impacts adipocyte biology and immune cell migration, a direct link between the HIV lifecycle and the severity of the induced fibrosis has not been described.

### Pharmacologic Parameters

The adipose toxicity of the first generation of ARVs (notably in *in vitro* assays) has been extensively described (Koethe, 2017). Nevertheless, the pharmacokinetics and intracellular metabolism of ARVs in adipocytes have not been extensively characterized. It is known that NRTIs and PIs accumulate in murine and human adipocytes *in vitro* (Janneh et al., 2003, 2010; Vernochet et al., 2005; Couturier et al., 2018) but only two *in vivo* studies (Dupin et al., 2002; Couturier et al., 2018) have reported on ARV concentrations in the AT of HIV-infected patients. Further studies of ARV penetration in AT are needed, and it remains to be established whether or not the tissue concentrations of the various ARVs in AT are therapeutic.

Indeed, the penetration of ARVs into AT is influenced by several physiochemical and pharmacokinetics factors, including the blood perfusion rate, molecular size, ionization state, plasma protein binding, lipophilicity, and efflux/influx transporter affinity.

The perfusion rate varies considerably from one tissue to another. AT is a poorly vascularized compartment with a low perfusion rate [approximately 200 mL/min or 0.025 mL/g tissue) (Derendorf et al., 2019)],which might limit the diffusion of ARVs. However, all of the ARVs except enfuvirtide are small molecules, and so can penetrate AT more easily. Given that only unbound, non-ionized drugs can cross cell membranes, the degree of binding to plasma proteins and the pKa are likely to influence AT penetration. It should be borne in mind that an ARV’s ionization state can differ from one tissue to another as a result of the local pH. In contrast to other tissues, penetration into AT did not appears to be related to the protein binding of ARVs (Dupin et al., 2002; Couturier et al., 2018). For example, most NRTIs penetrate genital and colorectal tissue extensively, notably as a result of their low plasma protein binding (<0.7–49%). In contrast, PIs, non-NRTIs, and integrase strand transfer inhibitors bind readily to plasma proteins (except for indinavir, nevirapine, and raltegravir in each of the three classes, respectively); >90% of the drug molecules are bound to albumin or alpha-1 acid glycoprotein, which thus may partly limit their access to genital and colorectal tissues (Trezza et al., 2015). Lipid solubility appears to be the most important factor in AT penetration. Hence, the relatively hydrophilic NRTIs (such as abacavir, lamivudine, emtricitabine, and tenofovir) are rarely or not detected in AT cells (Dupin et al., 2002; Couturier et al., 2018). In contrast, the more hydrophobic dolutegravir penetrates into AT in HIV-infected patients (Couturier et al., 2018). Lastly, the ARVs’ affinity for efflux or influx transporters [such as the ATP-binding cassette (ABC) and solute carrier transporter families] and the presence of transporters in AT may influence drug penetration. Although these transporters have been well characterized in various tissues (Kis et al., 2010), only one study of AT has been published; it found that P-glycoprotein (ABCB1) was expressed on pre-adipocytes and limits the accumulation of zidovudine (Janneh et al., 2010).

Other important factors include the complex structure of adipocyte tissue and the relationships between adipocytes and other cells. In SIV-infected macaques, Damouche et al. (2015) showed that CD4 T cells are distal to blood vessels in AT – implying that ARVs have to cross additional barriers if they are to reach infected immune cells in AT. Couturier et al. (2018) reported that the *in vitro* accumulation of tenofovir-diphosphate (the intracellular, active form of tenofovir) was low in CD4 T cells and was associated with increased HIV production.

The low levels of ARVs in AT and the corresponding impact on HIV replication suggest that AT is a pharmacological sanctuary in which high tissue concentrations of ARVs are required to suppress local HIV replication.

## Consequences of at Infection

Special focuses have been obviously given to the metabolic consequences of fat tissue infection that will be only briefly discussed in this review [see the excellent review on the subject (Desruisseaux et al., 2007; Koethe, 2017; Couturier and Lewis, 2018; Godfrey et al., 2019)]. Works are still ongoing notably to decipher the respective impact of the local viral infection and antiretroviral treatment. The question is even more relevant with the recent description of weight gain in patients (and notably women) treated with integrase inhibitors (Norwood et al., 2017; Bourgi et al., 2019; Kerchberger et al., 2019). However, more recent data suggest an even broader impact of fat tissue infection, potentially affecting systemic secondary immune responses and contributing to the accelerated aging, two defects commonly associated with chronic HIV infection.

### Metabolic Alterations Associated With Viral Persistence in AT

In treatment-naïve patients infected with HIV, we observed a number of metabolic alterations: a decrease in the amount of AT (Visnegarwala et al., 2005), AT redistribution (Madge et al., 1999), changes in adipogenic markers (Giralt et al., 2006; Vidal et al., 2012), and mitochondrial damage (Garrabou et al., 2011). Experiments in SIV-infected macaques have shown that chronic viral infection is associated with the presence of small adipocytes and elevated fibrosis – suggesting a specific impact of HIV infection on AT (Gorwood et al., 2019). Secreted viral proteins (such as Tat, Nef, Vpr, and gp120) have also been shown to influence the AT, although their secretion within the AT has never been observed. In a mouse model, Vpr induced metabolic changes in AT (Agarwal et al., 2013). Tat and Nef induced a profibrotic phenotype in adipose-derived MSCs, and Nef altered adipogenesis (Gorwood et al., 2019). The effect of Tat on adipogenesis is subject to debate (Cotter et al., 2011; Díaz-Delfín et al., 2012; Gorwood et al., 2019). The impact of Tat and Nef on MSCs has been studied extensively. Tat and Nef induce premature senescence in MSCs; Nef inhibits autophagy by binding to Beclin1, whereas Tat activates nuclear factor kappa B and this increases inflammation (Beaupere et al., 2015). Tat has been implicated in inflammation and elevated expression of pro-inflammatory cytokines in the AT (Díaz-Delfín et al., 2012), and activates nuclear factor kappa B in MSCs and CD4 T cells (Fiume et al., 2012). Taken as a whole, these studies suggest that HIV has a role in metabolic impairments in AT. Furthermore, various classes of ARVs (NRTIs, PIs, and now integrase strand transfer inhibitors) have long been implicated in the modification and redistribution of AT – suggesting that ART and HIV have an additional and/or synergistic impact.

### Immune Effects of Viral Persistence in AT

In the cynomolgus macaque model of intravenous infection with SIVmac 251, major changes in the CD4 T cell compartment in SAT and VAT were not observed; the CD4 T cell counts were unchanged, suggesting that limited depletion occurs in AT (Damouche et al., 2015). The main change was a significantly elevated CD8 T cell count. The specificity of CD8 T cell accumulation in AT remains to be evaluated, notably with regard to the recruitment of HIV-specific CD8 T cells. Changes in CD4 and CD8 T cell counts have been reported in ART-treated HIV-infected patients (Wanjalla et al., 2019). Given the putative role of AT as a site of memory T cell accumulation for lymphoid secondary immune responses, the nature of the change in the T cell compartment needs to be determined. One can reasonably hypothesize that HIV’s targeting of AT has both metabolic and immunologic consequences, with impaired memory responses against other pathogens (and not just HIV).

In contrast to the changes observed in a context of obesity, viral persistence does not appear to greatly modify the macrophage count. Phenotypically, the macrophages shift toward an M1 profile but less so than in a context of obesity. This intermediate profile suggests that the local inflammation associated with HIV infection differs from the “metabolic inflammation” associated with obesity. Similarly, viral persistence does not lead to great changes in the level of pro-inflammatory cytokine secretion. Overall, HIV-associated inflammation in AT may differ qualitatively and/or quantitatively from that one observed in the extreme context of obesity. The characteristics of AT in HIV infection vs. obesity have recently been nicely reviewed (Wanjalla et al., 2018).

### Accelerated Aging

Infection by HIV is thought to lead to the premature aging of AT, although further investigation is required. For example, the prevalence of comorbidities usually associated with aging (such as cardiometabolic disease) and the trunk fat accumulation phenotype are more prevalent in ART-treated HIV-infected patients (Caron-Debarle et al., 2010). Risk factors include HIV infection itself, and the duration and type of ART. The pathophysiologic mechanisms are poorly understood, although several factors are likely to be involved. ECM remodeling, inflammation, and redistribution have been observed in the AT of HIV-infected patients treated with first-generation ARVs (Bastard et al., 2002; Lagathu et al., 2005, 2007, 2017; Caron et al., 2007; Caron-Debarle et al., 2010; Béréziat et al., 2011). Interestingly, normal aging is also associated with fibrosis, inflammation, and AT redistribution (Tchkonia et al., 2010). Thus, one can hypothesize that HIV led to the premature aging of AT in these patients, and had cardiovascular and metabolic consequences. Immunosenescence has also been observed during chronic HIV-infection; this promotes immune activation and inflammation, which in turn might enhance HIV persistence and accelerate aging in a positive feedback loop (Appay and Sauce, 2008).

## Perspectives

### The Immune Properties of Adipose Tissue

Due to the physical size of AT and its critical location close to immune sites (i.e., interface sites or lymphoid sites), a better understanding of the tissue’s local and systemic properties is essential. Both metabolic and infectious signals may change the homeostasis of AT and then directly impair the effectiveness of secondary immune responses. A growing body of evidence points to an anti-infectious role for AT. Research in this field may highlight novel mechanistic links between obesity and susceptibility to infections.

Another feature of AT is its potential modulation by pharmacologic modulators of metabolism. Indeed, AT is doubtless a valuable model in which strategies aimed at correcting viral-induced defects and/or reducing the size of the viral reservoir can be evaluated.

### Therapeutic Strategies Based on Metabolic Pathways

At present, ART usually controls HIV replication in infected people. Nevertheless, cellular and tissue reservoirs of HIV are always present; it is still impossible to cure HIV and eradicate the virus from the body completely. The size of the HIV reservoir depends on several factors, including the time interval between the infection and the initiation of ART, the CD4/CD8 ratio, and the level of inflammation (even in patients on ART). To reduce systemic and local inflammation, pravastatin is already being used to treat HIV-infected patients (Toribio et al., 2017). Another possible treatment option is metformin, an antidiabetic, senolytic drug that might promote metabolic function in the AT, decrease inflammation, and modulate T cell activation (Moyo et al., 2014). Metformin’s effects have been studied in HIV-infected people with diabetes, and the drug is currently being tested clinically in non-diabetic HIV-infected patients (Routy et al., 2019). Another therapeutic strategy for controlling the HIV reservoir involves targeting immunometabolic checkpoints; metformin targets the 5′-adenosine monophosphate-activated protein kinase and the mammalian target of rapamycin (mTOR) (Routy et al., 2015), and the monoclonal antibody pembrolizumab targets PD-1 on infected CD4 T cells. This PD-1 blockade was initially used in cancer treatment but has been shown to reduce CD4 T cell HIV reservoirs *ex vivo* (Evans et al., 2018). Other immune checkpoint inhibitors (such as anti-CTLA4, mTORC1/2 inhibitors) used primarily to treat cancer might also be of value in reducing HIV reservoirs and persistence (Adashek et al., 2019). Two main strategies for reducing reservoirs and persistence are currently being investigated. Firstly, HIV “shock and kill” strategies are based on treatment with latency reversal agents (LRAs) and then immune clearance through cytopathic effects or immune-mediated responses. The HIV proteins Tat and Nef can be used as LRAs for latent CD4 T cells (Kuang and Brockman, 2018; Tang et al., 2018), whereas Nef inhibitors could be used as HIV-specific immune adjuvants for curative vaccines (Dekaban and Dikeakos, 2017). Anti-Bcl-2 drugs can also target and kill HIV-latent T cells, and thus direct reduce the HIV reservoir (Cummins et al., 2017). Secondly, the “deep latency” strategy adopt the opposite approach; proviral HIV is silenced so that it cannot rebound (Elsheikh et al., 2019). Lastly, recent studies have isolated several neutralizing monoclonal antibodies from HIV-infected patients – opening up the use of broadly neutralizing antibodies (bnAbs) as a potentially curative treatment for HIV (Possas et al., 2018). It has been suggested that bnAbs can control HIV replication and reactivate latent cells, although the use of these antibodies to specifically target HIV-1 in humans constitutes a major challenge (Shen et al., 2019). These cutting-age therapeutic strategies are currently under development. However, it remains to be seen whether these therapies can reach all the cellular and tissue reservoirs and, in particular, all the infected cells within the AT and other HIV sanctuaries (i.e., the central nervous system, spleen, and LNs).

## Conclusion

It is now well established that HIV infects AT, although the respective effects of the virus and ARVs on AT remain to be evaluated. This infection probably has more consequences than initially supposed. AT’s metabolic, immune, and regenerative properties mean that HIV infection triggers multiple defects in regenerative and/or immune functions. Regarding the mechanisms involved in HIV’s infection of (and then persistence in) AT, it is important to note that many pathogens are now known to persist in AT – suggesting the existence of an intrinsic mechanism that strongly favors their persistence. HIV persistence in AT may depend on various mechanisms; some may be intrinsic to the biology of AT, whereas others may be more closely related to virus-induced perturbations. Importantly, the AT’s metabolic activity may provide a means of therapeutically reducing or even eradicating the viral load in AT. A growing number of studies have demonstrated the effectiveness of metabolic approaches to immune modulation; this may be particularly relevant for decreasing the HIV reservoir in AT.

## Author Contributions

All authors, by their respective field of expertise (immunology, pharmacology, metabolism, and infectiology) contributed to the writing of the article.

## Conflict of Interest

The authors declare that the research was conducted in the absence of any commercial or financial relationships that could be construed as a potential conflict of interest.
